# Extraction of *Opuntia dillenii* Haw. Polysaccharides and Their Antioxidant Activities

**DOI:** 10.3390/molecules21121612

**Published:** 2016-11-24

**Authors:** Heng Li, Qingxia Yuan, Xianjiao Zhou, Fuhua Zeng, Xiangyang Lu

**Affiliations:** 1 College of Bioscience and Biotechnology, Hunan Agricultural University, Changsha 410128, China; liheng80@126.com; 2 School of Life Science and Technology, Lingnan Normal University, Zhanjiang 524048, China; zhouxianjiao@163.com (X.Z.); zzengfuhua@163.com (F.Z.); 3 School of Pharmacy, South-Central University for Nationalities, Wuhan 430074, China; qingxiayuan@mail.scuec.edu.cn; 4 Engineering Center of Resource Plant, Zhanjiang 524048, China

**Keywords:** *Opuntia dillenii* Haw., polysaccharide, extraction, antioxidation, characterization

## Abstract

Use of natural polysaccharides in medicine and food has wide interest in research. In this study, we extracted and purified some polysaccharides from cactus *Opuntia dillenii* Haw. (ODP). Some preliminary functions of these products were characterized. Under the optimal purification conditions, the yield of ODP extracted from the 2–4 month-old *Opuntia dillenii* Haw. (T-ODP) was 30.60% ± 0.40%, higher than that of ODP from the 5–10 month-old materials (O-ODP) (18.97% ± 0.58%). The extracted ODP was purified by DEAE sepharose fast flow anion exchange and Sephacryl S-400 chromatography with four fractions obtained (ODP-Ia, ODP-Ib, ODP-IIa and ODP-IIb). Analysis with UV-vis chromatography indicated that ODP-Ia and ODP-IIa were relatively homogeneous molecules with a molecular weight of 339 kD and 943 kD, respectively. Results of infrared spectroscopy indicated that ODP, ODP-Ia, and ODP-IIa were acidic polysaccharides. Further, the antioxidant activity against DPPH (1,1-diphenyl-2-picrylhydrazyl) radical, hydroxyl radicals, and superoxide radical in vitro demonstrated that the T-ODP exhibited higher antioxidant activity than the O-ODP, and the purified fraction (ODP-Ia) was superior to the ODP. These results will offer a theoretical basis for further research on the structure-function relationship of ODP and the rational utilization of *Opuntia dillenii* Haw.

## 1. Introduction

In recent years, much attention has been focused on polysaccharides isolated from natural sources such as bacteria, fungi, algae, and plants [1,2,3]. The wide range of biological activities and relatively low toxicities of these polysaccharides are the main reasons for the increased interests in this class of molecules [4,5]. Therefore, discovery and evaluation of new polysaccharides as new safe compounds for functional foods and medicines have become a hot research topic.

*Opuntia dillenii* Haw. is a cactus belonging to the Cactaceae family, widely grown in tropical and subtropical regions, including the south of China and Taiwan [6]. This plant has been consumed as food, natural coloring, sweetener, and forage. Traditionally, it has been utilized as a folk medicine in many countries [6,7]. The water soluble mucilage obtained from opuntia was found to be a neutral polysaccharide (*Opuntia dillenii* Haw. polysaccharides, ODP) that has many pharmacological functions, such as antihyperlipidemic, antioxidant, antidiabetic, immunostimulatory effects, and promotion of wound healing [5,8]. They may be potentially used as novel drug food supplements.

The current commonly used extraction methods have the disadvantage of producing a low extraction yield, although there have been studies on the optimization of the extraction technology for ODP [9,10]. The hot water extraction technology is still the classic and the main extraction method used to obtain the polysaccharides due to its convenience, low cost, and high activity [11,12,13]. At present, no information is available regarding the comparison of extraction yield and the antioxidant activities of ODP from different growth stages of the plant. In the present study, 2–4 month-old *Opuntia dillenii* Haw. was used as the raw material for the first time, in order to improve the extraction yield of ODP. The polysaccharides were extracted using the hot water extraction method and extraction conditions were optimized using an RSM (Response Surface Method) with a three-level, three-variable Box-Benhnken. The purification, the molecular weight, and the antioxidant activity of ODP, which may play a very important role in the characterization of polysaccharides, were studied.

## 2. Results and Discussion

### 2.1. Optimization of Extraction Conditions for ODP

**Figure 1:**
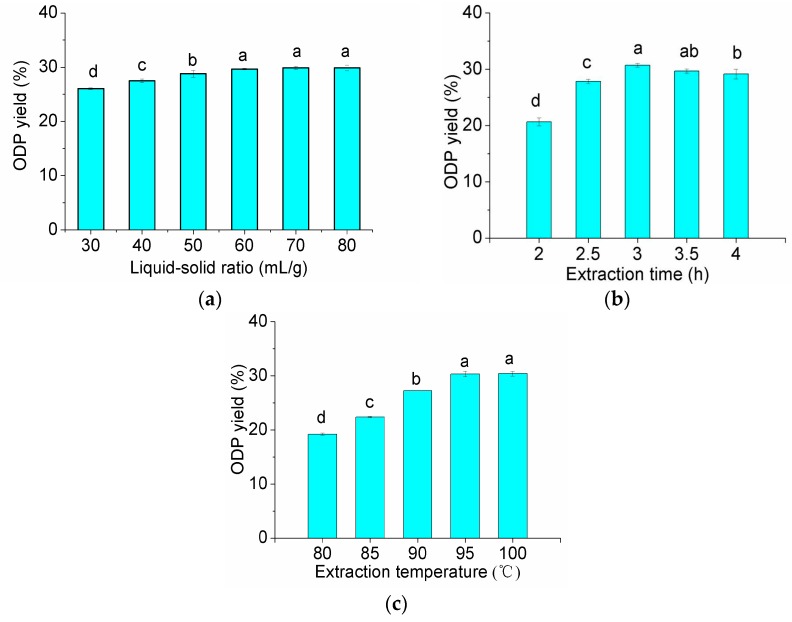
Effect of the different extraction parameters on the extraction rate of ODP (**a**–**c** are effects of liquid-solid ratio, extraction time and extraction temperature on the extraction rate of ODP, respectively). In the figure, different letters above the bars indicate that the values differ significantly at *p* < 0.05 (ANOVA followed by SNK-q test).

According to the data shown in Figure 1, liquid-solid ratio of 60 (mL/g), extraction time of 3 h and extraction temperature of 95 °C were the preferred extraction conditions. The RSM with a three-level, three-variable Box-Benhnkenthen was designed to determine the optimal extraction conditions for the highest extraction yield of ODP.

The regression equation (Equation (1)) was built by applying multiple regression analysis on the experimental data. The equation described an empirical relationship between the ODP yield and those test variables (A: Extraction temperature; B: Extraction time; C: Liquid-solid ratio) in coded units.

Y = 31.29 + 3.51A + 2.94B + 2.48C + 0.94AB − 0.65AC − 2.86BC − 5.54A^2^ − 3.65B^2^ − 4.16C^2^ (1)

As shown in Table 1, there is a good concordance of the predicted ODP yield values calculated from the regression equation with the experimental values, with a high total determination coefficient (R^2^) of 0.9908 (Table 2). The closer this value to 1, the better the fit between the model and the experimental data [14]. The Adeq precision of the model was 25.145. As a general rule, the fit rate of the predicted values and the experimental values can be explained well if its Adeq precision is greater than 4 [15].

According to the results of the analysis of variance, the model had a very low *p*-value (*p* < 0.01), indicating that the model was highly significant. A high *p*-value (*p* = 0.9522) on the lack of fit indicated that the failure of the model was not significant. These results suggest that the equation was adequate. The coefficient of variation (CV) was relatively low (3.39%), suggesting a high precision of the experiments, and a reasonable reproduction of the experiments can be carried out. Statistical testing of the independent variables was listed in Table 2. The data showed that all the linear coefficients (A, B, C), quadratic term coefficients (A^2^, B^2^, C^2^), and across product coefficients (BC) significantly affected the ODP yield (*p* < 0.05). Meanwhile, the extraction temperature (A) was the major factor affecting ODP yield with a very low *p*-values (*p* < 0.01), followed by extraction time (B) and liquid-solid ratio (C).

**Figure 2:**
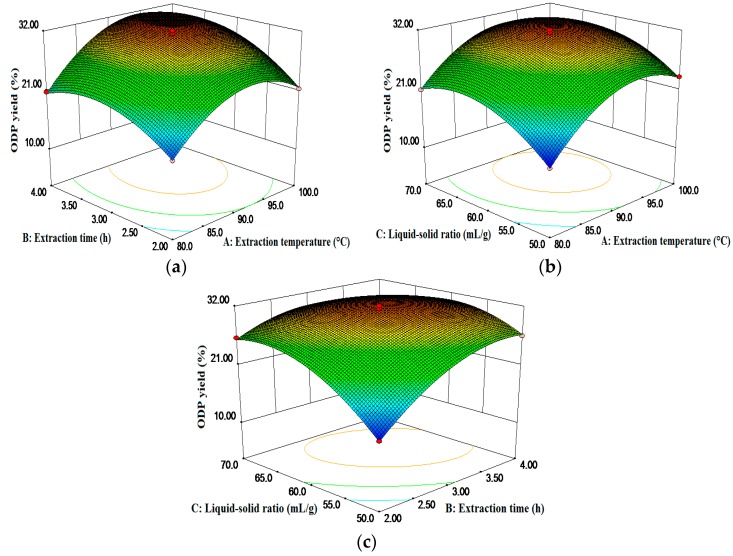
Response surface plots showing the predicted values for the ODP yield: the effects of two variables on the response ODP yield (%) with the other variable fixed at 0.

The three-dimensional graphical representations of the relationship between the independent and dependent variables, called the response surface and the contour plots, and the results of the ODP yield affected by the extraction temperature, the extraction time, and the liquid to solid ratio were presented in Figure 2. The figures demonstrated an elliptical contour plot, indicating that there was a perfect interaction between the independent variables, while a circular contour plot would indicate otherwise. The elliptical contour plot shown in Figure 2c indicated that the mutual interactions between the liquid-solid ratio (C) and the extraction time (B) were significant. The results are in agreement with Table 2.

By employing the Design-Expert software, the optimal values for the tested variables were as follows: extraction temperature of 100 °C, extraction time of 3.51 h, and liquid-solid ratio of 60.4 mL/g. To validate the stability of the optimal conditions, three verification experiments were performed under the optimal conditions mentioned above. The results indicated that the experimental ODP yield (30.60% ± 0.40%) was almost equal to the predicted ODP yield (30.29%), demonstrating the stability of the optimal conditions.

### 2.2. Comparison of Extraction Results of T-ODP and O-ODP

The extraction yield and the purity of T-ODP under optimal conditions were significantly higher than those of O-ODP (*p* < 0.05). The protein content showed no significant differences between T-ODP and O-ODP (*p* > 0.05). It is possible that polysaccharides in *Opuntia dillenii* Haw. accumulate rapidly in the early growing stage and that the lignin and cellulose content increased gradually afterwards (Table 3). Therefore, 2–4 month-old *Opuntia dillenii* Haw. is better suited for ODP extraction.

### 2.3. Homogeneity and Molecular Weight Analysis

**Figure 3:**
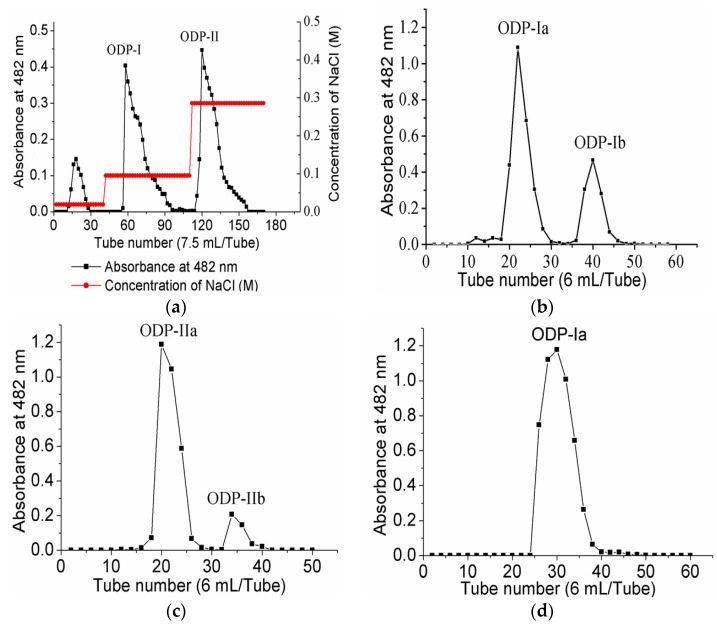

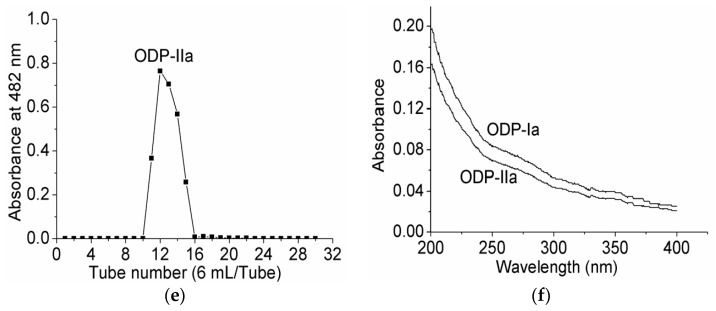
Chromatogram elution curves and UV-vis spectra of different ODPs. (**a**) DEAE-Sepharose fast-flow chromatogram elution curve of ODPs; (**b**) Sephacryl S-400 gel chromatogram elution curve of ODP-I; (**c**) Sephacryl S-400 gel chromatogram elution curve of ODP-II; (**d**) Sephacryl S-400 gel chromatogram elution curve of ODP-Ia; (**e**) Sephacryl S-400 gel chromatogram elution curve of ODP-IIa; (**f**) UV-vis spectra of ODP-Ia and ODP-IIa.

Upon purification by DEAE sepharose fast flow anion exchange column chromatography, ODP was found to contain two separate fractions, ODP-I and ODP-II (Figure 3a). The ODP-I and ODP-II fractions were further purified by Sephacryl S-400 column chromatography, which provided two fractions each, ODP-Ia and ODP-Ib, ODP-IIa and ODP-IIb (Figure 3b,c), respectively. The recovery rates of ODP-Ia, ODP-Ib, ODP-IIa and ODP-IIb based on the amount of ODP were 39.9%, 5.01%, 32.5%, and 14.47%, respectively. As shown in Figure 3d,e, ODP-Ia and ODP-IIa both gave a single and symmetrical peak in the Sephacryl S-400 column chromatograms, indicating that they were homogeneous polysaccharides and chromatographically pure. The regression equation that was obtained using Ve/V_0_ as the abscissa and log (Mr) as the ordinate was Ve/V_0_ = 2.474 log (Mr) + 15.767 and R^2^ = 0.9356 Ve, V_0_, and Mr means column calibration volume, column void volume, and molecular weight, respectively. According to the equation, the average molecular weight of ODP-Ia and ODP-IIa were 339 kD and 943 kD, respectively.

The UV-vis spectra of ODP-Ia and ODP-IIa are shown in Figure 3f. These UV-vis spectra suggested there is no optical absorption peaks at 260–280 nm. The results indicated that ODP-Ia and ODP-IIa did not contain protein or nucleic acid, since proteins and nucleic acids absorb light at 260 and 280, respectively [16].

### 2.4. FT-IR Spectroscopy

**Figure 4:**
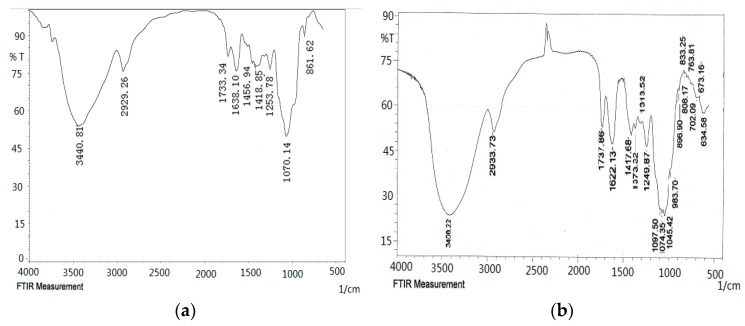

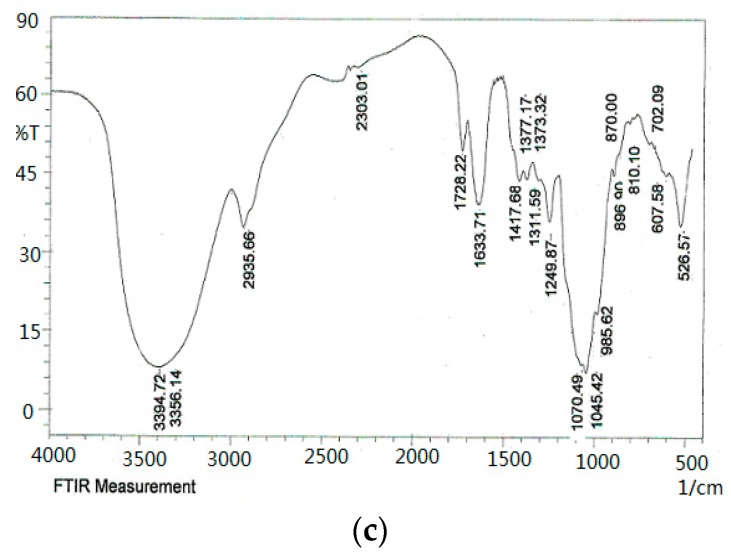
FT-IR of different ODPs. (**a**) ODP; (**b**) ODP-Ia; (**c**) ODP-IIa.

As shown in the FT-IR spectra (Figure 4), the ODP, ODP-Ia, and ODP-IIa have the typical polysaccharide absorptions. The absorption peaks of polysaccharide purified fractions ODP-Ia and ODP-IIa were roughly the same, and there were some differences with the ODP. The strong and broad absorption peaks at 3440.81 cm^−1^ (ODP), 3408.22 cm^−1^ (ODP-Ia), and 3394.72 cm^−1^ (ODP-IIa) were characteristic of the hydroxyl (OH) stretching of the glycan backbone. Peaks at 2929.26 cm^−1^ (ODP), 2933.73 cm^−1^ (ODP-Ia) and 2935.66 cm^−1^ (ODP-IIa) were attributed to alkane C–H stretching. The absorption peaks around 1733.34, 1638.10, and 1418.85 cm^−1^ of ODP, 1737.88, 1622.13, and 1417.68 cm^−1^ of ODP-Ia, 1728.22, 1633.71, and 1417.68 cm^−1^ of ODP-IIa indicated the presence of uronic acids, which suggested that ODP, ODP-Ia, and ODP-IIa were uronic acid-rich polysaccharides [17,18]. Each particular polysaccharide had peaks in the 1000–1200 cm^−1^ region. The peaks within this region are characteristic of the stretching vibrations of (C-OH) side groups and the (C-O-C) glycosidic bond vibrations.

Further, there were some special absorption peaks on ODP-Ia and ODP-IIa. The peaks at 896 cm^−1^ (ODP-Ia and ODP-IIa) were thought to be characteristic of β-anomeric carbon, indicating that ODP-Ia and ODP-IIa contained mainly β-type glycosidic linkages [19]. On the other hand, the peak at 861 cm^−1^ (ODP) is typical of the dominant α-configuration in pyranose form, indicating that ODP contained mainly α-type glycosidic linkages [20].

### 2.5. Antioxidant Activity of ODP

**Figure 5:**
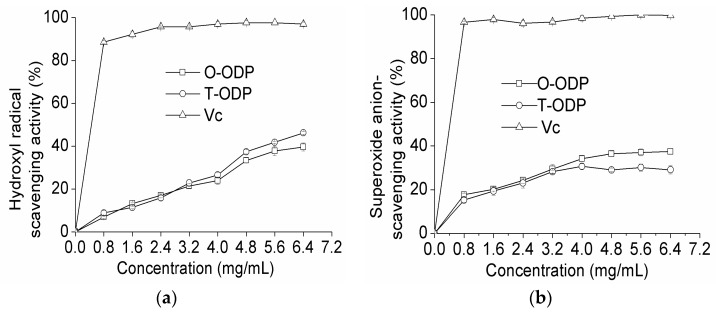

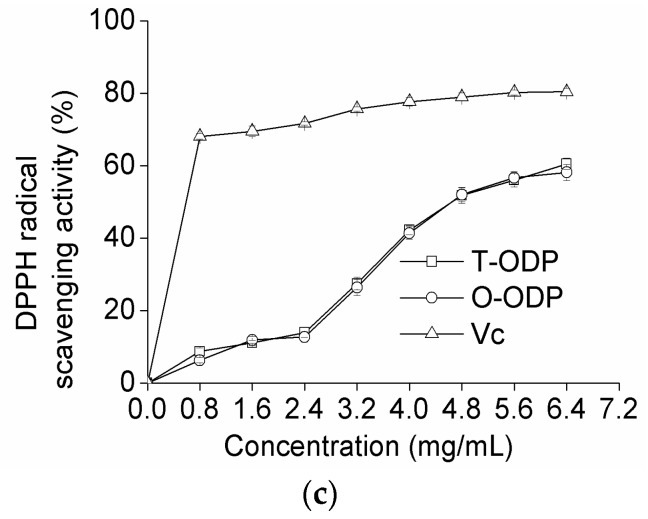
Scavenging effects on hydroxyl radical (**a**); superoxide radical (**b**); and DPPH radical (**c**) of T-ODP and O-ODP. T-ODP and O-ODP were extracted as described in Materials and Methods. Vc: ascorbic acid.

Hydroxyl radicals are well known as the most reactive free radicals. They react readily with most biomacromolecules including DNAs, proteins, lipids, and carbohydrates in living cells, and induce severe damage to the adjacent biomolecules [21]. As shown in Figure 5a at 0.8 mg/mL, T-ODP and O-ODP showed scavenging abilities of 6.92% and 8.86% on hydroxyl radicals, respectively. However, at 6.4 mg/mL, the scavenging effects increased to 39.62% and 46.20% for T-ODP and O-ODP, respectively. Scavenging abilities of the polysaccharides on hydroxyl radicals were in a concentration-dependent manner. However, at the same concentration level, the scavenging abilities of T-ODP and O-ODP on hydroxyl radicals were significantly lower than that of ascorbic acid.

As shown in Figure 5b, T-ODP and O-ODP exhibited no significant scavenging effect on superoxide radical. Nevertheless, for both T-ODP and O-ODP, the scavenging effect was concentration dependent. At 6.4 mg/mL, the scavenging activity of T-ODP was 37.44%, which was significantly higher than that of O-ODP (29.12%). Additionally, at all tested concentrations, and independently of how old it was and whichever the fraction used, the radical scavenging activity of ODP was lower than that of ascorbic acid used in this study.

The free radical of DPPH assay is a stable and widely used method to evaluate the free radical scavenging ability of natural compounds [22,23]. This method is based on the reduction of the absorbance of a DPPH ethanol solution at 517 nm in the presence of a proton-donating substance [24]. In this experiment, the DPPH free-radical scavenging effects of T-ODP, O-ODP, and Ascorbic acid (Vc) were measured, and the results are shown in Figure 5c. Both T-ODP and O-ODP showed strong scavenging effect against DPPH radical in a dose-dependent manner in all concentrations studied; the scavenging ratios at high concentrations of T-ODP and O-ODP (6.4 mg/mL) were 60.48% and 58.13%, respectively, and statistics analysis indicated that there was no significant difference between T-ODP and O-ODP. Nevertheless, the inhibiting abilities of T-ODP and O-ODP were both lower than that of ascorbic acid.

As shown in Figure 5, the difference of the radical scavenging activity between T-ODP and O-ODP was visible at 6.4 mg/mL. Therefore, the concentration of 6.4 mg/mL was selected for further analyzed. Table 4 depicted the radical scavenging activity of T-ODP and the various fractions at 6.4 mg/mL. T-ODP, ODP-Ia and ODP-IIa showed the highest scavenging activity (*p* < 0.05), while, ODP-Ib and ODP-IIb showed the weakest scavenging activity (*p* < 0.05), especially ODP-IIb. ODP-Ia showed slightly higher scavenging effect than ODP-IIa (*p* < 0.05). Several reports postulated that the smaller the molecular weight, the higher antioxidant effect for polysaccharides [24,25]. According to our results, the molecular weight followed the order: ODP-Ia < ODP-IIa, and the antioxidant effect followed the order: ODP-Ia > ODP-IIa, which is in accordance with that reported by Asker et al. [24] and Yu et al. [25]. However, while T-ODP showed lower radical scavenging activity than ODP-Ia, it was higher than ODP-I and ODP-II. It implied that molecular weight is not the only factor affecting the antioxidant effect [26]. Many variables affect the antioxidant properties of polysaccharides, including their monosaccharide composition, conformation, degree, and distribution pattern of sulfate, among others [27,28,29].

## 3. Materials and Methods 

### 3.1. Chemicals and Material 

Fresh tender *Opuntia dillenii* Haw. cladodes of 2–4 month and 5–10 month specimens were collected from Donghai Island, Zhanjiang, Guangdong Province, China. Coomassie brilliant blue G250 and l-(+)-Arabinose (≥90.0%) were obtained from Fluka Chemie (Buchs, Switzerland). DPPH radical, DEAE sepharose fast flow anion exchange and Sephacryl S-400 were purchased from Sigma (Washington, WA, USA). All other chemicals and reagents used were of analytical grade.

### 3.2. Extraction of ODP 

The preliminary treatment of *Opuntia dillenii* cladodes of uniform shape and maturity was performed as described previously [8]. Each dried pretreated sample (1.0 g) was extracted using hot water at a designated ratio of raw water volume to material weight of 30–80 mL/g. The extraction was performed at 80–100 °C for 2–4 h. The extracts were centrifuged at 1810× *g* for 15 min, at 4 °C. The supernatants were collected and concentrated using a vacuum rotary evaporator (LABOROTA 4003-control, Schwabach, Germany) at low pressure (60 kPa). Proteins in the solution were removed using the trichloroacetic acid method [30]. The supernatants collected by centrifugation at 1810× *g* for 15 min, at 4 °C, with the pH adjusted to neutral were added to 95% ethanol (triple the volume of the solution) at 4 °C. Precipitates were collected by centrifugation at 1810× *g* for 15 min, at 4 °C and were re-dissolved in deionized water to the volume equal to that of the original supernatant before precipitation. The resulting solutions were dialyzed in dialysis tubing (MWCO 3500 Da, Spectrum Laboratories, Laguna Hills, CA, USA) against distilled water for three days at 4 °C. Water was replaced twice a day to remove monosaccharides, oligosaccharides, and other compounds of low molecular weights. After the dialysis, the volumes of the polysaccharide solutions were recorded, and the ODP contents were determined using the phenol-sulfuric acid method, with arabinose as the standard [31]. The solution was precipitated again using 95% ethanol. The precipitates were collected by centrifugation at 1810× *g* for 15 min at 4 °C. They were washed with anhydrous alcohol and lyophilized to obtain ODP powder. The protein content of the ODP powder was determined using the Coomassie brilliant blue method with bovine serum albumin as a standard [32]. The polysaccharide purity was determined using the phenol-sulfuric acid method and calculated using the following formula: polysaccharide purity (%) = measured weight (g)/ODP sample weight (g).

### 3.3. Optimization of the Extraction Conditions for ODP 

The extraction parameters were optimized using RSM with a three-level, three-variable Box-Benhnken (BBD, software Design-Expert v.8.0, Stat-Ease, Minneapolis, MN, country). Extraction temperature (°C, A), Liquid-solid ratio (mL/g, B), Extraction time (h, C), were chosen for independent variables. The range and center point values of these variables were based on the results of single-factor experiments and are presented in Table 5. ODP yield (%, *w*/*w*) was selected as the response for the 17 experimental points shown in Table 2. In order to minimize the effects of unexpected variability in the observed responses, the 17 experimental points were carried out randomized.

### 3.4. ODP Extraction from Tender and Old Opuntia dillenii Haw. 

T-ODP and O-ODP were obtained from the tender (2–4 months old) and the old (5–10 months old) *Opuntia dillenii* Haw. cladodes respectively. Each dried pretreated sample (1.0 g) was extracted using the optimal extraction conditions. The extraction yield and purity of the extracted ODP were determined, and the protein content in the ODP was also measured as above.

### 3.5. Purification of the Polysaccharide

An amount of 125 mg of ODP powder was dissolved in 10 mL of phosphate buffer (0.05 mol/L, pH 5.9). After intensive dissolution, 6 mL of the suspension was loaded onto a DEAE-Cellulose anion-exchange (DEAE sepharose fast flow anion exchange) column (2.6 cm × 60 cm) pre-equilibrated with phosphate buffer (0.05 mol/L, pH 5.9), and then eluted successively with NaCl gradients (0.02, 0.1 and 0.3 mol/L) prepared in the same buffer, at a flow rate of 2.5 mL/min. Each fraction (7.5 mL) was collected and assayed at 490 nm for polysaccharide by phenol-sulfuric acid method [31]. The eluents corresponding to a single polysaccharide peak were pooled together and lyophilized after being dialyzed against distilled water for 48 h at 4 °C. Dried fractions were dissolved in phosphate buffer (0.015 mol/L, pH 5.9). One milliliter of the solution was loaded onto a Sephacryl S-400 gel filtration column (1.6 cm × 100 cm) and eluted with phosphate buffer (0.015 mol/L, pH 5.9) at a flow rate of 0.4 mL/min. Each fraction of 3 mL was monitored and processed as described above.

### 3.6. Characterization of ODP and Its Purified Fractions

#### 3.6.1. Molecular Weight 

The molecular weight of the ODP components were determined by a gel filtration method using Sephacryl S-400. Column void volume (V_0_) measurement was performed by passing 1.0 mL blue dextran (200,000 Da, 1.0 mg/mL) through the column (1.6 cm × 100 cm) and run with a potassium phosphate buffer (pH 5.9) at a flow rate of 0.4 mL/min, and then by measuring its effluent volume by phenol-sulfuric acid method. Column calibration volume (Ve) was done by running standard dextran samples (T2000, T500, T70, T40, and T10) in the same procedure. The regression equation was obtained using Ve/V_0_ as the abscissa and log (molecular weight) as the ordinate. The Vt of ODP components was measured using the same method. The molecular weights of the ODP components were determined from the regression equation, using their Vt/V_0_ value.

#### 3.6.2. UV-Vis spectrophotometric Analysis

The UV-vis spectrophotometric analysis was carried out using the method of Yang [33] with one modification. Each purified polysaccharide components (1 mg) was dissolved in 10 mL of distilled water. The absorbance of each sample solution was determined over the range of 200 to 400 nm with 0.2 nm line interval, using a UV-2102 PC UV-visible spectrophotometer (Unico, Shanghai, China). The distilled water was scanned in the same way as the blank control.

#### 3.6.3. FT-IR Spectroscopy Analysis

The infrared spectra of ODP, ODP-Ia, and ODP-Ib were determined using a Fourier transform infrared spectrophotometer (Bruker, Madison, WI, USA). The samples were ground with spectroscopic grade potassium bromide (KBr) powder and then pressed into a 1 mm pellet for FT-IR determination between wave number range of 4000–500 cm^−1^ [34].

### 3.7. In Vitro Antioxidant Activity

#### 3.7.1. Scavenging Activity on DPPH Radical

DPPH free radical scavenging activity of ODP was assessed using the method of Bamdad [35] with some modifications. Specifically, 2.0 mL of 0.2 mmol/L DPPH ethanol solution was added to 2.0 mL of ODP solution of various concentrations (0, 0.8, 1.6, 2.4, 3.2, 4.0, 4.8, 5.6, 6.4 mg/mL). The mixture was shaken well and incubated for 30 min in the dark at room temperature; the absorbance was measured at 517 nm against a blank (DPPH replaced by ethanol) with ascorbic acid as a positive control. The scavenging effect on DPPH free radical was calculated using the formula:

DPPH scavenging effect (%) = [1 − (A_i_ − A_j_)/A_0_] × 100% (2)

where A_0_ is the absorbance of the control (50% ethanol instead of ODP solution); A_i_ is the absorbance of the sample; A_j_ is the absorbance of the sample under identical conditions as A_i_ with 50% ethanol instead of DPPH solution.

#### 3.7.2. Scavenging Activity on Hydroxyl Radical

The hydroxyl radical scavenging activity of ODP was measured according to the method of Xie [36] with a minor modification. Specifically, 1.0 mL of 0.15 mol/L sodium phosphate buffer (pH 7.4), 1.0 mL of 0.75 mmol/L 1,10-phenanthroline, 1.0 mL of 0.75 mmol/L FeSO_4_ solution, and 1.0 mL water were added to 1.0 mL of sample solution at different concentrations (0, 0.8, 1.6, 2.4, 3.2, 4.0, 4.8, 5.6, 6.4 mg/mL). Finally, 1.0 mL of H_2_O_2_ (0.01%, *v*/*v*) was added. The reaction mixture was incubated at 37 °C for 60 min. The absorbance of the mixture was measured at 536 nm. Ascorbic acid was used as the positive control. The hydroxyl radical scavenging activity was calculated according to the equation:

Scavenging activity (%) = (A_s_ − A_i_)/(A_0_ − A_i_) × 100% (3)

where A_s_ is the absorbance of the reaction mixture with the sample; A_i_ is the absorbance of the reaction mixture with the sample replaced by an equivalent volume of distilled water; A_0_ is the absorbance of the reaction mixture with the sample, and H_2_O_2_ replaced by an equivalent volume of distilled water. 

#### 3.7.3. Scavenging Activity on Superoxide Radical

The scavenging activity for self-oxidation of 1,2,3-trihydroxybenzene of all the samples was performed using the method of Marklund [37] with a minor modification. In our experiment, 4.5 mL Tris-HCl (50.0 mmol/L, pH 8.2) was mixed with 4.2 mL water and 1.0 mL of sample solution at different concentrations (0, 0.8, 1.6, 2.4, 3.2, 4.0, 4.8, 5.6, 6.4 mg/mL) and incubated for 20 min at 25 °C. The samples were then removed from the incubator and 0.3 mL of 25 mmol/L preheated 1,2,3-trihydroxybenzene was added to the solution. The mixture was quickly mixed thoroughly. Its absorbance was determined at 325 nm at intervals of 30 s for 4 min. The scavenging activity for self-oxidation of 1,2,3-trihydroxybenzene was determined as follows:

Scavenging activity (%) = (A − A_s_)/A × 100% (4)

where A is the change speed of absorbance of the 1,2,3-trihydroxybenzene and water; A_s_ is the change speed of absorbance of the 1,2,3-trihydroxybenzene and sample, and the unit was calculated as the absorbance increase per minute.

### 3.8. Statistical Analyses

All the experiments were performed in triplicate. The data were expressed as the mean ± standard deviation (SD) and analyzed using one-way analysis of variance (ANOVA), followed by SNK-q test. Differences were considered to be statistically significant (*p* < 0.05) or highly significant (*p* < 0.01). All statistical analyses were carried out using SPSS 13.0 for windows (SPSS, Chicago, IL, USA).

## 4. Conclusions

In this study, we successfully optimized the extraction yield of ODP by RSM test. The optimal conditions for ODP extraction were found to be as follows: extraction temperature 100 °C, extraction time 3.51 h, and liquid-solid ratio 60.4 mL/mg. Under these optimal conditions, the extraction yield and the purity of T-ODP were significantly higher than those of O-ODP (*p* < 0.05). The protein content, however, showed no significant differences between T-ODP and O-ODP (*p* > 0.05). Therefore, 2–4 month-old *Opuntia dillenii* Haw. was selected as the effective raw material. Purification results showed that T-ODP contained four fractions ODP-Ia, ODP-Ib, ODP-IIa, and ODP-IIb. ODP-Ia and ODP-IIa showed to be homogeneous polysaccharides with a molecular weight of 339 kD and 943 kD, respectively. FT-IR Spectroscopy results showed that ODP, ODP-Ia, and ODP-IIa were uronic acid-rich polysaccharides. T-ODP and O-ODP showed an excellent antioxidant activity on DPPH radical, and a good activity against hydroxyl radical and superoxide radical, and ODP-Ia exhibited the highest antioxidant ability. Based on these results, we believe that ODP could be a source of natural antioxidants and food supplement in the pharmaceutical and medical industries. However, the apparent differences of antioxidant ability existed among the ODP fractions. Hence, further investigations should be considered to obtain more understanding of the effect of chemical structure on the antioxidant ability.

## Figures and Tables

**Table 1:** Box-Behnken design matrix and the response values for ODP yield.

No.	A	B	C	ODP Yield (%)	
	Extraction Temperature (°C)	Extraction Time (h)	Liquid-Solid Ratio (mL/g)	Experimental	Predicted
1	90	3	60	30.45	31.29
2	90	3	60	31.45	31.29
3	80	2	60	16.53	16.59
4	90	2	50	15.27	15.20
5	100	4	60	29.55	29.48
6	80	3	50	14.95	14.95
7	90	3	60	33.05	31.29
8	90	4	70	25.97	26.04
9	90	4	50	26.54	26.80
10	90	3	60	30.4	31.29
11	90	3	60	31.11	31.29
12	90	2	70	26.14	25.88
13	100	3	50	23.46	23.26
14	80	2	60	21.45	21.72
15	80	3	70	21.02	21.21
16	100	3	70	26.93	26.93
17	80	4	60	20.85	20.58

**Table 2:** The variance analysis for the response surface experiment.

Source	SS	Ms	*F*-Value	Prob > F	Significance
Model	542.07	60.23	83.93	<0.0001	**
A	98.28	98.28	136.95	<0.0001	**
B	69.15	69.15	96.36	<0.0001	**
C	49.20	49.2	68.57	<0.0001	**
AB	3.57	3.57	4.98	0.0609	
AC	1.69	1.69	2.36	0.1688	
BC	32.72	32.72	45.59	0.0003	**
A^2^	129.39	129.39	180.31	<0.0001	**
B^2^	56.2	56.2	78.32	<0.0001	**
C^2^	72.81	72.81	101.47	<0.0001	**
Residual	5.02	0.72			
Lack of fit	0.37	0.12	0.11	0.9522	
Pure error	4.65	1.16			
Cortotal	547.09				

** Significant at 0.001 level; Std. Dev = 0.85, R^2^ = 0.9908; Mean = 25.01; Adj R^2^ = 0.9790; C.V.% = 3.39; Pred R^2^ = 0.9759; Adeq precision = 25.145.

**Table 3:** Extraction results of T-ODP and O-ODP (x ± s, *n* = 6).

Type	ODP Yield (%)	Purity (%)	Protein Content (%)
2–4 month-old *Opuntia dillenii* Haw.	30.60 ± 0.40 ^a^	85.32 ± 0.32 ^a^	0.65 ± 0.09 ^a^
5–10 month-old *Opuntia dillenii* Haw.	18.97 ± 0.58 ^b^	57.99 ± 0.89 ^b^	0.87 ± 0.12 ^a^

In each column, different letters above the bars indicate that the values differ significantly at *p* < 0.05 (ANOVA followed by SNK-q test).

**Table 4:** Radical scavenging activity of ODP fractions at 6.4 mg/mL (%, $\bar{x}$ ± s, *n* = 6).

Samples	Antioxidant Activity (%)		
	DPPH Radical	Hydroxyl Radical	Superoxide Radical
T-ODP	54.45 ± 1.46 ^c^	40.78 ± 0.56 ^c,d^	36.88 ± 0.99 ^c^
ODP-I	46.17 ± 1.67 ^d^	39.83 ± 0.90 ^c,d^	36.50 ± 0.89 ^c^
ODP-Ia	58.44 ± 1.37 ^b^	45.69 ± 0.92 ^b^	43.71 ± 1.30 ^b^
ODP-Ib	15.29 ± 0.56 ^f^	12.47 ± 0.30 ^e^	13.37 ± 0.41 ^e^
ODP-II	33.92 ± 1.14 ^e^	39.07 ± 1.46 ^d^	30.01 ± 0.21 ^d^
ODP-IIa	45.55 ± 0.89 ^d^	41.47 ± 1.18 ^c^	37.19 ± 0.57 ^c^
ODP-IIb	8.80 ± 0.74 ^g^	0.84 ± 0.12 ^f^	5.22 ± 0.12 ^f^
Vc	80.06 ± 0.85 ^a^	96.35 ± 0.98 ^a^	99.13 ± 0.09 ^a^

In each column, different letters above the bars indicate that the values differ significantly at *p* < 0.05 (ANOVA followed by SNK-q test).

**Table 5:** Independent variables and the levels used for the BBD design.

Independent Variables	Levels		
	−1	0	1
A: Extraction temperature (°C)	80	90	100
B: Liquid-solid ratio (mL/g)	50	60	70
C: Extraction time (h)	2	3	4
